# Idiopathic Clubbing Confined to Lower Limb Digits and Idiopathic Pulmonary Fibrosis: An Unusual Association

**DOI:** 10.1155/2012/684285

**Published:** 2012-10-22

**Authors:** Rahul Magazine, Ranjan Shetty, Umesh Goneppanavar, Aswini K. Mohapatra

**Affiliations:** ^1^Department of Pulmonary Medicine, Kasturba Medical College, Manipal University, Karnataka, Manipal 576104, India; ^2^Department of Cardiology, Kasturba Medical College, Manipal University, Karnataka, Manipal 576104, India; ^3^Department of Anaesthesiology, Kasturba Medical College, Manipal University, Karnataka, Manipal 576104, India

## Abstract

A 62-year-old housewife presented to the chest outpatient department with a history of exertional breathlessness of four-month duration. On general physical examination, clubbing of toes was present with sparing of fingers. Chest examination revealed bilateral basal end inspiratory fine crepitations. A diagnosis of idiopathic pulmonary fibrosis was made on the basis of clinical, spirometric, and high-resolution computed tomography findings. Extensive evaluation could not reveal any cause for the differential clubbing. The unusual distribution of clubbing in a clinical condition, such as idiopathic pulmonary fibrosis, where generalized clubbing is expected can lead to a diagnostic confusion. This can lead to a further burden of investigations on the patient as clubbing being a significant finding cannot be ignored.

## 1. Introduction

Clubbing is the bulbous enlargement of the distal segment of digits [1]. It has been described in association with pulmonary, cardiac, neoplastic, gastrointestinal, infectious, and endocrine disorders [2]. It can also occur in healthy people as an idiopathic condition or a hereditary trait [3]. Clubbing is usually present in digits of all the limbs. However, in certain conditions, it can be confined to one or two limbs [2].

Idiopathic pulmonary fibrosis (IPF) is a nonneoplastic pulmonary disease in which scar tissue is formed within the lungs in the absence of any known provocation. The prevalence is estimated to be 13.2 to 20.2 cases per 100,000, with about half of the cases having clubbing [4]. To the best of our knowledge, idiopathic clubbing confined to toes, by itself or in association with idiopathic pulmonary fibrosis, has not been described in the literature.

## 2. Case Report

**Figure 1:**
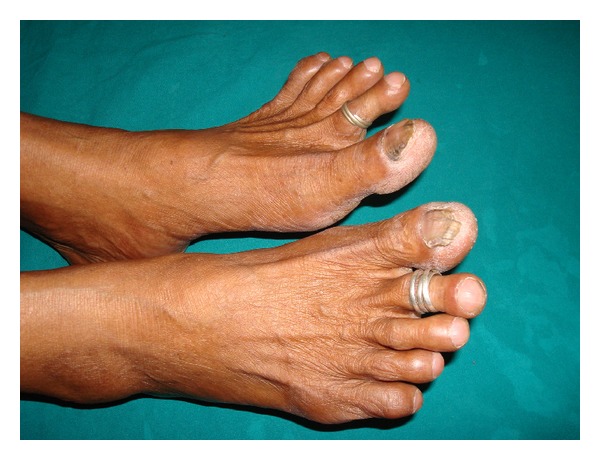
Clubbing of digits of feet with no edema or skin changes at ankles.

**Figure 2:**
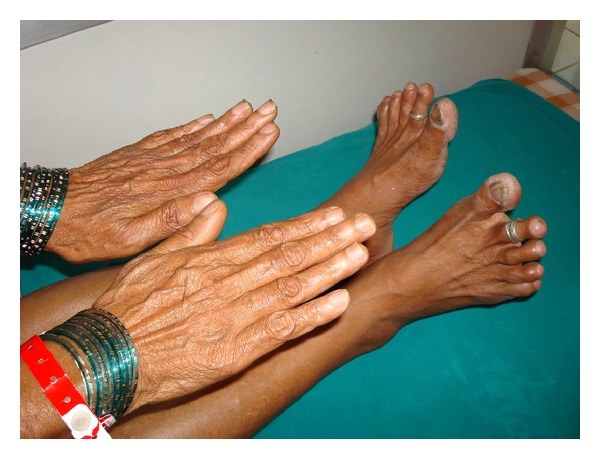
Absence of clubbing in digits of hands with no skin changes or edema at wrists while the clubbing is present in the toes.

**Figure 3:**
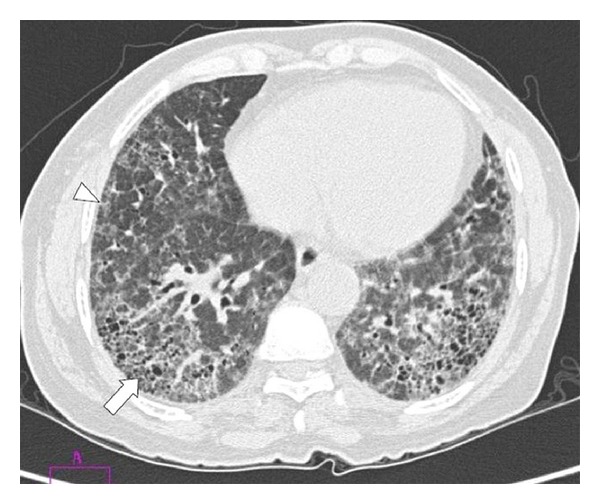
HRCT demonstrating interstitial thickening (arrow head) and honeycombing (arrow) in basal regions of lung.

A 62-year-old housewife presented to the chest out patient department with a history of breathlessness on exertion of four-month duration. She did not give any history of exposure to drugs or environmental inhalational agents. There was no history of chest pain, palpitations, ankle swelling, syncope, or gastrointestinal symptoms. She was a nonsmoker and did not consume alcohol. There was no history of clubbing in the family, and she was not aware of the presence of clubbing in her lower limb digits. Review of her previous medical records did not reveal the presence of clubbing or any major illness in the past. She had two children, and both were born by normal vaginal delivery. She had attained menopause at the age of 45 years. On general physical examination, pulse rate was 80/min, regular, good volume and all the peripheral pulses were palpable. Respiratory rate was 15/min and blood pressure 130/80 mmHg, and she was afebrile. SpO_2_ at rest was 98–100% on room air. Clubbing of all the toes in both feet was present, but none of the fingers were involved (Figures 1 and 2). Presence or absence of clubbing was definitely established by calculating the ratio between diameters at the bases of the nails and at the distal interphalangeal joints of all 10 digits of upper and lower limbs separately [5]. The sum of individual digit ratios was found to be more than 10 in lower limbs. Swelling or skin changes were clearly absent around the wrists and ankles (Figures 1 and 2). There were no features suggestive of connective tissue or endocrine disorder. Chest examination revealed bilateral diffuse end inspiratory fine crepitations predominantly at the lung bases. Examination of abdomen and other systems did not reveal any abnormality. Hemogram showed a hemoglobin of 12.5 gm/dL, total leucocyte count 9400/mm^3^ with 61% neutrophils, 37% lymphocytes, and 2% eosinophils. Erythrocyte sedimentation rate was 12 mm in the first hour. Blood biochemical examination and routine urine analysis were normal. The thyroid and liver function tests were normal. The workup for connective tissue disorders such as anti-nuclear antibody and rheumatoid factor was negative. Serological testing for human immunodeficiency virus was negative. Sputum smear for acid fast bacilli was negative. Blood and sputum cultures were sterile. Spirometry showed a restrictive pattern. Chest roentgenography revealed haziness in both lower zones. High-resolution computed tomography (HRCT) was showing interstitial thickening and honey combing in basal regions (Figure 3). Contrast CT did not reveal any evidence of aneurysm involving thoracic or abdominal aorta. Ultrasonography of the abdomen, fiberoptic bronchoscopy- and electrocardiogram were normal. Her echocardiogram did not show any intracardiac shunt. Contrast echocardiography study was also normal, suggesting no evidence of pulmonary arteriovenous fistula. Examination of her siblings and children did not reveal the presence of clubbing.

## 3. Discussion

Clubbing of toes with sparing of fingers has been described in association with infected abdominal aortic prosthesis, infected abdominal aortic aneurysm, and patent ductus arteriosus [5, 6]. However, history, physical examination, and echocardiography in this case ruled out the presence of all three of these conditions. It may be difficult to differentiate clubbing of toes from normal variations due to effect of shoes and posture, based on visual examination alone. Hence, the clubbing confined to lower limb digits was assessed using objective methods for determining clubbing, as described above in the case report [5, 6]. Usually, the patients will be unaware of the presence of clubbing which was the case in this patient [2]. Hence, it was difficult to ascertain whether the differential clubbing was present since birth or developed later on in life. However, there was no evidence of clubbing in the family nor was there any mention of clubbing in her medical records, which made hereditary clubbing an unlikely diagnosis. Moreover, hereditary clubbing is an isolated finding and is more common in males than females [3].

A surgical lung biopsy is needed for the definitive diagnosis of idiopathic pulmonary fibrosis, but it was not considered necessary in this case in view of the typical clinical and HRCT findings [4, 7]. Cases of idiopathic pulmonary fibrosis are associated with clubbing, but differential clubbing has a different set of causes which do not include idiopathic pulmonary fibrosis. Hence, this association leads to diagnostic difficulty. However, systematic exclusion of the known causes of differential clubbing confined to toes, and also the fact that idiopathic pulmonary fibrosis could not explain the development of differential clubbing, made us conclude that in this case there was incidental occurrence of idiopathic differential clubbing. Extensive search of literature revealed that there are two features that make this case interesting. Firstly, the idiopathic nature of differential clubbing confined to toes and secondly its association with idiopathic pulmonary fibrosis. 

 If the coexisting clinical condition cannot explain the presence of clubbing or its distribution, then it can lead to diagnostic confusion, further workup, and hence additional burden of investigations on the patient. However, clubbing being an important clinical finding cannot be ignored and needs to be investigated.
